# Health service changes to address diabetes in pregnancy in a complex setting: perspectives of health professionals

**DOI:** 10.1186/s12913-017-2478-7

**Published:** 2017-08-03

**Authors:** R. Kirkham, J. A. Boyle, C. Whitbread, M. Dowden, C. Connors, S. Corpus, L. McCarthy, J. Oats, H. D. McIntyre, E. Moore, K. O’Dea, A. Brown, L. Maple-Brown, On behalf of the NT Diabetes in Pregnancy Partnership

**Affiliations:** 1 Wellbeing and Preventable Chronic Disease, Menzies School of Health Research, Darwin, NT 0811 Australia; 2 Monash Centre for Health Research and Implementation, School of Public Health and Preventive Medicine, Monash University, Melbourne, Australia; 3 Diabetes Education Unit, Royal Darwin Hospital, Darwin, Australia; 4 Health Services and Planning, Sunrise Health Service Aboriginal Corporation, Katherine, Australia; 5 Top End Health Services, Northern Territory Department of Health, Darwin, Australia; 6 Womens Health, Danila Dilba Butji Binnilutlum Health Service Aboriginal Corporation, Darwin, Australia; 7 Melbourne School of Population and Global Health, University of Melbourne, Melbourne, Australia; 8 Mater Medical Research Institute, University of Queensland, Brisbane, Australia; 9 Aboriginal Medical Services Alliance Northern Territory, Darwin, Australia; 10 Population Health Research, University of South Australia, Adelaide, Australia; 11 South Australian Health and Medical Research Institute, Adelaide, Australia; 12 Department of Endocrinology, Royal Darwin Hospital, Darwin, Australia

**Keywords:** Health services, Healthcare delivery, Diabetes in pregnancy, Integration of care, Indigenous

## Abstract

**Background:**

Australian Aboriginal and Torres Strait Islander women have high rates of gestational and pre-existing type 2 diabetes in pregnancy. The Northern Territory (NT) Diabetes in Pregnancy Partnership was established to enhance systems and services to improve health outcomes. It has three arms: a clinical register, developing models of care and a longitudinal birth cohort. This study used a process evaluation to report on health professional’s perceptions of models of care and related quality improvement activities since the implementation of the Partnership.

**Methods:**

Changes to models of care were documented according to goals and aims of the Partnership and reviewed annually by the Partnership Steering group. A ‘systems assessment tool’ was used to guide six focus groups (49 healthcare professionals). Transcripts were coded and analysed according to pre-identified themes of orientation and guidelines, education, communication, logistics and access, and information technology.

**Results:**

Key improvements since implementation of the Partnership include: health professional relationships, communication and education; and integration of quality improvement activities. Focus groups with 49 health professionals provided in depth information about how these activities have impacted their practice and models of care for diabetes in pregnancy. Co-ordination of care was reported to have improved, however it was also identified as an opportunity for further development. Recommendations included a central care coordinator, better integration of information technology systems and ongoing comprehensive quality improvement processes.

**Conclusions:**

The Partnership has facilitated quality improvement through supporting the development of improved systems that enhance models of care. Persisting challenges exist for delivering care to a high risk population however improvements in formal processes and structures, as demonstrated in this work thus far, play an important role in work towards improving health outcomes.

## Background

Rates of diabetes are disproportionately higher among Indigenous populations internationally; with similar rates reported between Aboriginal populations in Australia and Canada, particularly among younger age groups [1–4]. Prevalence of diabetes in pregnancy (DIP), defined here to include both pre-pregnancy type 2 diabetes in pregnancy and gestational diabetes mellitus (GDM), has increased globally [5] alongside increases in prevalence of obesity and type 2 diabetes. There is a disproportionately high burden among Aboriginal and Torres Strait Islander women in Australia (here after respectfully referred to as Aboriginal in this paper), with 10 times the rate of pre-pregnancy type 2 diabetes and 1.5 times the rate of gestational diabetes of non-Aboriginal women [6]. Developing effective strategies to address the emerging challenges posed by DIP, including an increased risk of early-onset type 2 diabetes [7–9], are essential to improving health outcomes for Aboriginal people, including prevention of intergenerational impacts of diabetes at earlier ages in offspring of mothers with DIP [10–12].

The Northern Territory (NT) covers 1.3 million square kilometres, and 29.6% of the population of 245,100 are Aboriginal people, of whom 80% live outside the major city. In 2014, 36.3% of NT births were to Aboriginal mothers [6]. Twelve percent of all NT mothers were reported to have GDM, with a higher rate among Aboriginal (16%) than non-Aboriginal women (10%) [13]. The comprehensive primary health care system in the NT is complex. The size of health services are impacted by remoteness, diversity of clients and population size. While there are health clinics and outreach services in most rural and remote communities in the NT, access to specialist care is often limited [14]. To address the burden of DIP in Central Australia, a Diabetes Antenatal Care and Education clinic was established in 2003 as a central point of referral and management. However, challenges in delivering appropriate diabetes care to rural and remote women include: fragmented care, lack of connectivity between patient information systems in different services (a common challenge for health care in many settings) and remote workforce shortages [15]. Social determinants of health also impact the effectiveness of care delivered to remote dwelling Aboriginal people with challenges including food insecurity, poor housing, low education levels, social disadvantage and remoteness [16, 17]. Other issues include sustainability of primary health care, workforce retention, education policy and funding [18].

**Fig. 1:**
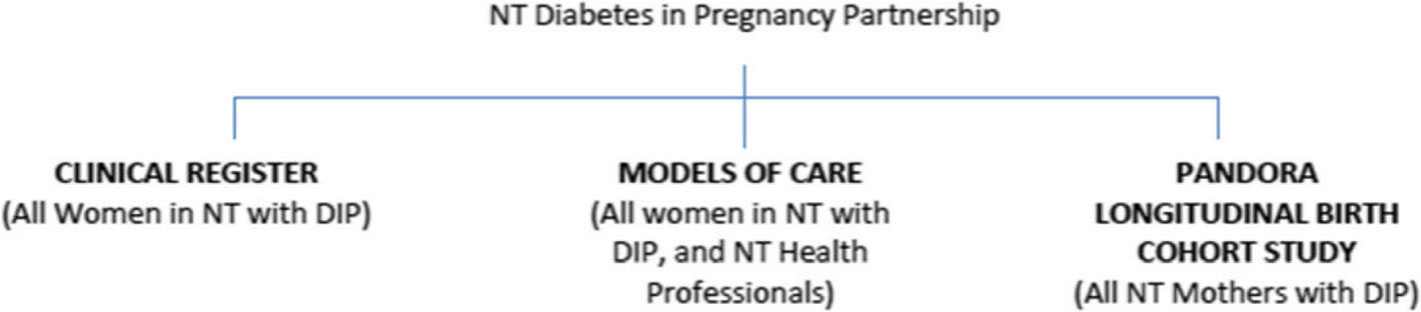
Partnership structure 2012–2016

In this context, the NT DIP Partnership (henceforth referred to as the Partnership) between researchers, policy makers and health organisations, including the Aboriginal Medical Services Alliance of the NT was developed to improve maternal and neonatal outcomes among women with DIP. The Partnership has three arms; the NT DIP Clinical Register established as an epidemiological and quality assurance tool; a longitudinal research study documenting the rates and outcomes of DIP [19]; and models of care (See Fig. 1). The models of care arm aims to review and enhance current systems and practices across the NT and is the focus of the current manuscript.

**Fig. 2:**
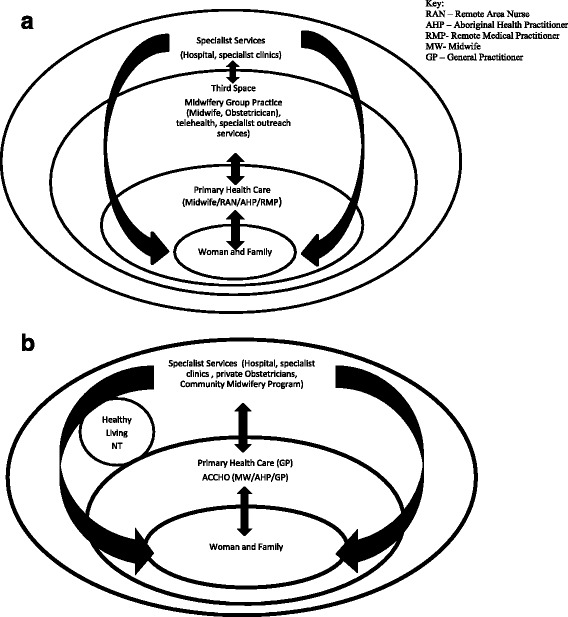
**a** Remote Models of Care. **b** Urban Models of Care

**Fig. 3:**
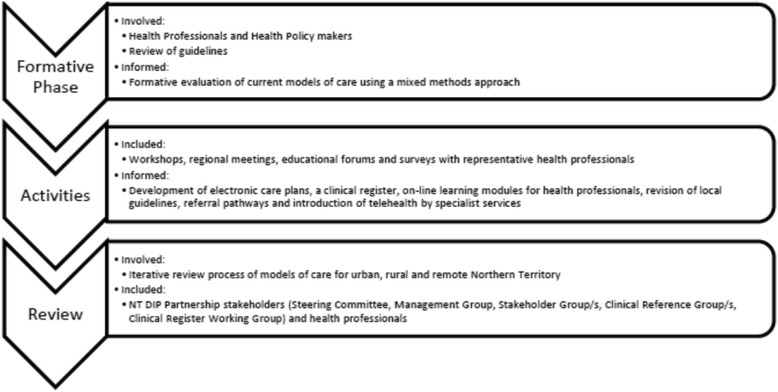
Process of developing models of care for DIP in the Northern Territory

Since establishment in 2011, the models of care structure essentially remains unchanged, continually aiming to progress connectivity and integration of systems and services (See Fig. 2a and b). In the remote model, continuity of care is offered to remote Aboriginal women by the Midwifery Group Practice from the antenatal through to post-partum period. Whereas all women in the urban setting are offered diabetes support through Healthy Living NT. Figure 3 outlines the work to refine models of care development. A Steering Group involved researchers and clinicians who met annually to review and plan models of care strategies. A primary focus of models of care has been on promoting communication and education through symposiums, workshops and reports (including opportunities for participation by teleconference for remote health professionals). Between 2011 and 2015, the Partnership ran annual fora, 15 workshops, 31 meetings, education programs (including researchers, clinicians, service delivery providers and policy makers) and distributed bi-annual reports and newsletters. Clinical register reports were circulated from 2013 as a quality improvement activity for models of care, including information about DIP prevalence and outcomes. Reports were discussed and implications for practice, management, communication and integration of care deliberated. Furthermore, Members of the Partnership contributed to writing local guidelines; including the Central Australian Rural Practitioners Association Standard Treatment Manual, and Women’s Business Manual [20], incorporating World Health Organisation (WHO) [21] recommendations. All activities have enabled information feedback to Partnership members, who raise issues and facilitate changes as part of routine practice.

To assess health service delivery for DIP at the start of the Partnership in 2012, health care professionals in the NT participated in three regional workshops (2011–2012) and a survey. Key issues were identified across five themes including: orientation and guidelines; education; communication; logistics and access; and information technology [22]. This paper aims to assess how the Partnership has addressed shortcomings of existing models of care as identified in its preliminary work [22]. Specifically, it uses a process evaluation focusing on health professional’s perceptions of quality improvement activities and enhanced models of care.

## Methods

### Partnership models of care

Since establishment in 2011, the Partnerships’ primary goal for the models of care work was to support implementation of evidence gaps in practice. This included promoting early testing of women, integration of primary and tertiary care, linking antenatal and diabetes care providers, improving communication strategies to link primary and tertiary service providers, develop integrated DIP care plans on existing IT systems, providing care according to current protocols and guidelines and provide relevant education and training for health practitioners.

### Focus groups evaluating models of care (MOC)

To better understand the impact of quality improvement processes from health practitioners involved in delivering DIP care, six focus groups were conducted (October 2015 – February 2016) in Alice Springs, Darwin and Nhulunbuy. To ascertain the strengths and weaknesses of current systems the Systems Assessment Tool (SAT) [23] was used as a framework to guide the focus group discussions. This tool was chosen based on its use in Australian Aboriginal primary health care centres and its aim to identify how to improve integration and functioning [24, 25].

The SAT has five main components: delivery system design; information systems and decision support; self-management support; links with community and other services; and organisational influence and integration. For the purpose of this study each focus group was guided by different components of the SAT to identify barriers and enablers of DIP models of care. Furthermore, the issues identified by Edwards (2014) are covered by the scope of the SAT, contributing to its suitability for this study.

Partnership networks were used to recruit individuals involved in care delivery to women with DIP from across the NT. Participation in focus groups was voluntary. Participants were (*n* = 49): six general practitioners and/or public health physicians, two obstetricians, one endocrinologist, nine midwives, four nurses (public health and chronic disease), 10 remote area nurses, eight diabetes educators, four allied health (dietician, nutritionist, health promotion), three Aboriginal health practitioners and two health professionals from undisclosed disciplines.

The four focus groups in Alice Springs were conducted at the same time by four facilitators, with components from the SAT separated between groups to obtain in-depth understandings from each. These focus groups occurred following an educational-symposium on chronic disease and an update on Partnership activities. Groups consisted of practitioners working with clients from similar regions (Central Australia, Arnhem Land, Darwin Region). The average duration of focus groups was 75 min (ranging from 73 to 81 min). Two focus groups were also conducted in Darwin (addressing all SAT components) and Nhulunbuy (addressing SAT components where more information was required, duration 152 and 184 min). With consent, all focus groups were audio-recorded, transcribed verbatim and coded in NVivo (version 10). Thematic deductive analysis (in line with pre-determined themes from earlier work [22]) was employed to determine the extent to which existing concepts, explanations, results and theories, as outlined in the SAT, were supported [26]. Data were analysed independently by two authors (RK and MD), a coding structure with themes and subthemes further developed, and analysis cross-checked for accuracy.

Ethics approval was gained from the Human Research and Ethics Committee (HREC) of the NT Department of Health and Menzies School of Health Research and the Central Australian HREC.

## Results

### Quality improvement processes

The Partnership has facilitated quality improvement processes for DIP care delivery across the NT (as outlined in Table 1). These are reported in line with initial themes identified by Edwards [22]. Most are related to care-coordination and reflect the increased opportunities for communication and strengthened networks through educational workshops and associated Partnership activities (including clinical register quality assurance activities). Since establishment, the network has expanded to involve active participation from health professionals in different regions. In 2011, three meetings were facilitated in the major centre increasing to seven meetings and symposiums in 2016, which were held in sites across the region. On-going support from the primary and tertiary health care sectors has been demonstrated through letters of support for extension of the Clinical Register and activities of the Partnership. Table 2 provides an overview of systemic changes that have occurred since the establishment of the Partnership.

**Table 1:** NT DIP Partnership activities

Barriers identified in 2012	Activities and impact of NT DIP Partnership
Communication * Breakdown between tertiary and primary health services referral pathway difficult, unsuitable specialist clinic times*	- Workshops and regional meetings resulted in an increased understanding of roles and priorities of different disciplines from tertiary and primary health care settings and increased contact between clinicians; created congenial relationships and enhanced case conferencing and discussion. - Engagement of clinicians in process of development of referral pathways resulted in an increased uptake of referral pathways and care plans.
Access * Lack of access for remote clients to specialist services (*e.g. *dietitians), food insecurity*	- Increased access to specialist services through telehealth and allied health outreach visits resulted in enhanced local health professionals’ knowledge. - Establishment of nutrition in pregnancy working group resulted in the development of nutrition education resources. - Partnership activities resulted in midwives taking on the role of commencing blood glucose monitoring with the women. All NT diabetes services are now able to provide home blood glucose monitoring equipment to women. Women are now more likely to have blood glucose profiles when attending appointments. Prior to this, women presenting for the first time at the antenatal clinic with a diabetes diagnosis, rarely came with a glucose monitoring profile.
Education * Health professionals reported knowledge gaps, no structured education available, resources for women not easily located*	- Partnership staff delivered workshops, education sessions and presentations at hospital for a grand rounds, Primary Health Network events, university undergraduates and conferences across the NT, facilitated by primary health and tertiary organisations. - On-line learning modules were developed for health professionals. - Investigators revised local guidelines to be in line with ADIPS and WHO, and incorporated them into Central Australian Rural Practitioners Association (CARPA) Standard Treatment Manual (6th Edition) and the Minymaku Kutju Tjukurpa – Women’s Business Manual (5th Edition). - Educational activities have heightened awareness of early detection of DIP leading to the development of a clinic in one Aboriginal Medical Health Service for women to attend to have an OGTT (either antenatal or post-partum). - Educational activities have resulted in an increased awareness of testing and reporting of DIP, with annual increases in numbers of women with DIP being reported by NT Midwives Data Collection.
Coordination and Transition of Care *Unsure of who was involved in management of women and who was responsible for co-ordinating the care. between primary and tertiary health services.*	- Patient Journey Modelling and educational partnership activities resulted in increased clinician contact which enhanced the coordination and transition of care. - Workshops and regional meetings resulted in specialist clinic time revised in order to suit assessment and treatment modalities with minimal ‘out of community’ time for the women. - Workshops and regional meetings resulted in care-coordination becoming part of clinical care at outpatient clinics with a meeting at the end of each clinic for multi-disciplinary team members to collaborate on a plan of care for complex cases. - Electronic care plans for diabetes in pregnancy were developed for use in primary health care. - The clinical register generates a weekly working list for monitoring the care coordination of women with diabetes in pregnancy at each hospital in the NT.

**Table 2:** Documented systems changes

Baseline (2012)	Current (2016)
Communication	
*Centralised specialist clinics in tertiary hospital* *Poor communication between services (with opportunities to improve use of telemedicine*)	- Systemic integration of telemedicine into health services - Regular case conferencing with remote clinics through telehealth - Clinical Register reports circulated quarterly with information around prevalence of DIP across regions - Improved referral pathways - Enhanced awareness by PHC clinicians of availability of hospital-based specialists for phone advice and case conferences
Access	
*Limited engagement of diabetes and allied health specialists in remote settings and limited access to these specialist services close to home for remote women*	- Regular outreach visits and telehealth by specialists (including dieticians) - Increased capacity of Primary Health Care clinicians to manage DIP in remote communities with support by phone/telehealth from hospital-specialists (hub and spoke model)
Education	
*Minimal DIP educational activities for Health Professionals* *Limited self-management educational resources available for women* *Limited access to glucose monitors*	- Regular DIP educational forums for Health Professionals (including a focus on preconception care and postpartum care) - DIP educational resource for women - Free access to glucose monitors
Coordination and Transition of Care	
*Remote clients required to travel to access specialist care*	- Specialist clinic times changed to better meet the needs of remote clients - Electronic care plans used in primary health care. - Multi-disciplinary collaboration at outreach meetings to coordinate care provided to complex cases
Clinical Guidelines	
*Use of a standard treatment manual, based on the Australian Diabetes in Pregnancy Society guidelines* *Different guidelines used in Primary Health Care and hospital and in different regions within the NT*	-Adoption of International Association of Diabetes in Pregnancy Study Groups and World Health Organisation guidelines (which have a lower threshold for diagnosis and earlier screening) -Guidelines were aligned between Primary Health Care and hospital to be consistent between guidelines and across all NT regions -Strong promotion of guidelines in education sessions

Focus groups identified the following improvements that have been facilitated by or resulted from activities of the Partnership (Table 3), reported in line with the initial themes of Edwards.

**Table 3:** Health Professional Focus Groups: additional data according to identified themes

*Orientation and guidelines*
*‘There’s so much expert knowledge that we have developed in the NT, most people actually don’t know when they first arrive.’*(Public Health Physician) *‘There has been involvement from the remote nutritionists […] more access to dieticians [resulting in] a real robustness around the remote midwives feeling comfortable about some basic messages.’* (Diabetes educator)
*Education*
*‘[We] are dealing with a population with the most complex health issues […] the most complex social issues. Of course, it is not going to be easy.’* (Public health physician) *‘[There is] so much more awareness out in the community now’.*(Diabetes educator)
*Communication*
*‘The trouble with midwifery coverage in Central Australia is that it is delivered by five different services all accountable to different line managers, all with different priorities and stuff like that. And no sort of overall clinical coordination.’* (Remote midwife)
*Logistics and Access*
*‘[Continuity of care is made] easier because [of] the [Aboriginal] Liaison Officer who […] actually go[es] out to some of the hostels in Alice Springs and bring[s] [clients] in.’* (Remote diabetes educator) ‘[*Aboriginal Health Practitioners are critical especially] if we don’t know where people are staying, or we don’t know if they are in [town], we do not have a clue how to get a hold of them to bring them in[to the clinic].’* (Remote diabetes educator) *‘[Since the commencement of the partnership] there’s been a really strong level of engagement which has been a very positive thing because it takes a few years of you know people getting new information and then looking at how they integrate that into their routine work practice and you know I suspect it may not be until actually after the end of the project that we really start to see perhaps the impact of what’s all the changes that [….] been putting into place.’* (Diabetes Educator)
*Information Technology*
*'A dedicated virtual clinic [like] tele-health [would overcome some problems with communication].* (Diabetes educator)
*Opportunities for further improvements in MOC*
*‘[A Care Co-ordinator would be useful] because you’ve got such a vast area of clinics with you know, who don’t have the resources of the midwife or people who know what they’re doing, I think that’s a great idea.’* (Remote outreach midwife) ‘*A health directory would be very very useful.’*(Remote general practitioner) ‘*We need a mechanism to take collective knowledge, so that when someone comes in new, [they don’t] have to wait until they’ve been here for seven years before they know who to refer to.’* (Public health physician)

#### Orientation and guidelines

Orientation of health professionals to the burden of DIP and reported adherence to guidelines contributed to quality improvement and improved care-coordination. Firstly, a comprehensive understanding of the burden of DIP for Aboriginal women was described among health professionals. This may be attributed to improved communication, educational activities and subsequent knowledge of DIP management. Many reported adhering to current guidelines, (the Central Australian Rural Practitioners Association Standard Treatment Manual and the Minymaku Kutju Tjukurpa – Women’s Business Manual). Health professionals also reported increased understanding of the urgency in treating these women appropriately.

Clinicians also reported an increase of women presenting with a diabetes management strategy initiated prior to review in a tertiary specialist clinic. This has been attributed to improved adherence to guidelines in community health centres, which may be related to increased awareness through quality improvement activities including workshops, forums, reports and newsletters. A public health physician stated:

*We tend to stay ahead of the game because of the population prevalence and so we move to things faster than we would in a general population.*

#### Education

Many participants referred to the Diabetes Antenatal Care and Education clinic as facilitating improved engagement with clients and access to integrated care. This clinic operates one day per week and is dedicated to reviewing women with DIP (also offering support for health professionals in the area). A remote midwife described its role of *‘tak[ing] care of [diabetic] women ante-natally and post-natally and […] get[ting] the information out’*. Another midwife said that *‘our women seem to know that if they have diabetes then Wednesday is the day that they come [for this clinic].’*

#### Communication

The Partnership has enabled a heightened awareness of DIP and a more collaborative approach to care. As described by a public health physician, it has improved communication between relevant health professionals and services through:

*…the regional meetings, the clinical reference group and […] one of the really important things there is the multi-disciplinary nature which […] has broken down those silos and given people permission to be talking on a more regular basis. In terms of staff morale, just thinking across everyone, staff morale actually seems very high with this […] Partnership.*

As expressed by a diabetes educator, it has facilitated health professionals *‘…understanding each other’s business better’*, and improved *‘collaboration between the midwife and the diabetes educator*’. As a quality assurance tool, the establishment of the clinical register has also had positive implications for improved communication between relevant stakeholders.

One of the most prominent issues inhibiting effective models of care is communication with multiple care providers, as described by a midwife:

*I find that the most tedious part of the practice – is actually communicating with all sectors these women are involved in.*

This is further implicated by health professionals often not knowing who to contact. Remote area nurses were quoted by a remote outreach midwife as saying *‘“who do I ring? […] I don’t know what to do”’*, and then *‘they ring the remote medical practitioner who is their program doctor, and that person is in Sydney (major city 3,934km from Darwin) and they’re not sure either.’*

It was suggested that this was not the case for staff who have been working in the area for some time. For one endocrinologist, *‘the pathways are clear’*, because, as supported by a public health physician, they have *‘been around for quite a while, and that [knowledge] just develop[s] with your networks over time.’* The Partnership has worked to develop clear referral pathways but focus group findings suggest further work is needed in this area to streamline and clarify information, particularly in the context of high staff turnover. In one focus group, the question was raised by a public health physician of whether or not a central hub that everyone could contact to access the key network would be helpful: *‘a person at primary health network and […] a website [health professionals could refer to]’*.

#### Logistics and access

The Partnership has also been successful in providing better access to specialist services, including to diabetes education, dietitians and access to glucose meters (Table 1). Collaboration between primary and tertiary health care professionals was also reported to have improved with both the establishment of the Partnership and other concurrent initiatives, including the Diabetes Antenatal Care and Education clinic and Midwifery Group Practice. Local health professionals reported an increase in the profile of Diabetes Antenatal Care and Education clinic occurring alongside the Partnership work. Furthermore, the Midwifery Group Practice, which assists continuity of care and the transition between primary and tertiary care for Aboriginal women from remote communities has contributed to improved access to care. Improved care-coordination can therefore also be attributed to enhanced engagement and access between different health sectors and services.

Other changes included improved access to hospital electronic records (i.e. Midwifery Group Practice commenced using hospital electronic health records), development of referral pathways (the antenatal clinic coordinator developed an enhanced method of referral to the antenatal clinic midwife), and improved care-coordination (including transition between primary and tertiary health services as reported by Midwifery Group Practice who have been facilitating this communication).

A Memorandum of Understanding was also endorsed by the Partnership with suppliers of glucose meters to provide free glucose metres to women with DIP. This enables all women to self-monitor, thereby encouraging early initiation of plans to manage hyperglycaemia.

However, persisting barriers to access and optimal integration are workforce shortages. Some of these challenges were acknowledged by a public health physician: *‘we will never have enough [health professionals] because we are still literally scratching the surface in terms of unmet need. And so it is about constantly re-prioritising, and re-focusing, and keeping everybody on track to make sure we are staying [on track].’*

Care delivery relies on individuals – particularly those who work hard, have local knowledge and relationships with the community. This model is not sustainable, because when this person leaves the system fails. For example, the sustainability of the clinic in Alice Springs was described by a remote midwife as being dependent upon the *‘great relationships’* staff have with individuals in remote clinics. The communication strategies of this clinic are *‘not actually embedded in practice’*, rather rely on individual knowledge and relationships, *‘that just needs that person to leave’* to jeopardise the model.

As such, working in the remote health context of the NT requires task shifting because many staff are required to operate at higher levels to account for the disease burden. Expectations of staff being adaptable and well informed to deal with complex situations may also influence the problem of *‘high staff turnover’*, as put by a remote midwife, which is problematic because *‘the next person who comes in starts to work on something that has already been existing’.*

While some discourses alluded to staff shortages, individuals or local systems being the cause of many issues related to models of care, others contradicted this and offer alternative explanations. A public health physician stated *‘it’s not the individuals. It’s actually about making sure we’re organised and systematic to get the best outcomes for the clients.’*

It was recognised that better collaboration with Aboriginal health team members would overcome some workforce issues, including; facilitating continuity of care, contributing to expert contextual knowledge and improving coordination of women’s care. A remote chronic disease nurse encourages the support of ‘*the Strong Women [senior Aboriginal women in Aboriginal communities whose role is to support younger women throughout their pregnancy]’.* She acknowledges that *‘you’ve got to work with the groups, with the family and use the community workers, your Aboriginal staff in the community, they’re the ones that know [clients] best.’*

Furthermore, the mobility of many Aboriginal people across the vast and sparsely populated NT can make it difficult to locate women and track the care they have been receiving, as a remote midwife described *‘…women start to move off of the radar because they are moving around.’* A remote general practitioner believes this creates challenges in maintaining continuity of care, where *‘…the faults are almost exclusively not the hospital [or] with the educators […], it’s just that we sort of drop the ball, we can’t find the person.’*

#### Information technology

Enhancing IT systems to improve communication and care-coordination was raised as a strategy to improving models of care. Telehealth consultations were introduced to improve access to specialist services for remote clients. In 2012 this involved teleconferencing and in 2014 video link up. A remote nurse said that the *‘weekly meetings [staff at her clinic have] with the Midwifery Manager [via telehealth] helps.’*

Furthermore, having two different electronic health record systems in the NT was raised as a barrier to care-coordination. It was suggested by a remote general practitioner that content *‘consistent with other clinics in the NT would be really useful’.* In addition, an Aboriginal health practitioner raised an issue with e-health in relation to staff turnover.

*‘we get regularly rung up by people who haven’t checked e-health so the staff turnover here with people not knowing or getting orientated on to e-health or not hav[ing] access is just so huge.’*

### Recommendations for improved care-coordination

Despite activities of the Partnership having contributed to developing patient pathways, several focus groups indicated the need for improved care coordination. One public health physician stated that *‘we need a traffic controller’* to overcome issues not only with referral and treatment pathways, but to better co-ordinate care particularly in instances such as that described by an urban midwife:

*[In] Central Australia when [the Aboriginal women] come into town, they just get lost. There’s no-one co-ordinating their care in town.*

In addition, data supported the need for clearly outlining who to call for advice in various regions, particularly important in the context of high staff turnover in remote clinics.

For example, an obstetrician said that a *‘list of the structure of what happens in the Royal Darwin Hospital (tertiary hospital)’* would be useful for staff in Arnhem Land (remote region 600 km away), including information around *‘what days the clinics are, what the intent is, who is there and who the contact people are for the clinics...’* While this has already been done for some sites, a diabetes educator reinforced *‘that there needs to be some place that you can actually ring for advice […] and you don’t have to tell someone how big [community] is.’*

## Discussion

The NT DIP Partnership was established to improve systems and services for DIP in the context of an Australian setting with global health challenges of socio-economic disadvantage, remoteness and a high risk Aboriginal population. In this interim evaluation of the Partnership we report three key findings related to quality improvement processes from the perspectives of health professionals’ involved in delivering care: enhancing health professional relationships; facilitating improvements to communication and integration of care; and, contributing to health professionals’ ability to operate at a higher level (including reported adherence to clinical guidelines). As this was the only intervention addressing DIP in the NT, the Partnership played a central role in promoting quality improvement processes. This manuscript did not aim to assess the effectiveness of the Partnership intervention in terms of quality and consistency of care, nor client perceptions of changes. Future work of the Partnership will report on these aspects of models of care.

We report that the quality improvement processes of the Partnership have enhanced relationships and communication throughout the region since 2012. Specifically, networks between health professionals have been strengthened and engagement and access across different health sectors and services improved. The role of the Midwifery Group Practice has assisted with client transition by working in the third space (community-based settings between the hospital and primary health care) [27], although it is limited to certain regions and transition continues to be a challenge for women elsewhere. Persisting barriers include factors associated with communication, care-coordination and transition. Communication is impeded by health services using different electronic medical information systems. These issues could be addressed at the systems level through better integration and coordination of health records and IT systems [28], although significant challenges remain in integration of IT systems between government, non-government and Community-Controlled sectors.

The second finding is that quality improvement activities promoting education and guidelines may have contributed to health practitioners’ ability to operate at a higher level. Health professionals, particularly those in remote settings, have described adopting practices appropriate to their context which may be partly in response to the poor health outcomes associated with DIP in the Aboriginal health context. Health professionals often reported exceeding the duties of their specified role to respond to the complex needs of their clients. An underlying expectation exists around staff capability, including adaptability and expertise, particularly in remote locations. A shift from traditional models of health care delivery to an integrated population health approach has been recommended by others, requiring appropriate workforce support (including education and training activities) [29, 30]. Task-shifting from physicians to non-physician healthcare workers, in combination with health system re-structuring, has been identified as one potential solution to improve access to healthcare in low and middle income countries [31] and is also a strategy used in Australian Aboriginal health. High performance may address some staffing issues (i.e. staff shortages) in the short term, however heavy workloads put staff at risk of burnout and are not sustainable [32]. Suggested opportunities for further improvements were to engage more Aboriginal Health Practitioners [33, 34] and Strong Women Workers [35] in this workforce. Partnership models involving Aboriginal Health Practitioners have been successful elsewhere [36–38].

Health professionals reported strong adherence to current clinical guidelines in their practice as was also reported by Edwards at Partnership commencement [22]. Clear guidelines for DIP screening, diagnosis and management are critical. While there is controversy internationally in relation to guidelines, this is not the case in the NT, with health care professionals reported following local guidelines (although this was not formally measured). Quality improvement activities of the Partnership (including workshops, newsletters and the clinical register) have improved communication between staff and relevant stakeholders, and assisted with the transfer of knowledge on updated guidelines and appropriate models of care.

Although care coordination systems have improved since the establishment of the Partnership, further improvements are required. Disjointed communication between systems and individuals is a persisting challenge and impacts upon continuity of care. One strategy to improve communication was that of a new position of central “care coordinator”. This may also overcome issues related to frequent staff turnover and individual knowledge loss. A serious commitment to creating a central repository (which includes educational resources and information about referral pathways) accessible to all health professionals is required. A feedback mechanism to make improvements to systems involved in care is also necessary. In line with earlier research, better integration and coordination of IT health records and IT systems is recommended and would provide an opportunity for improved access to specialist care and improved communication [22]. In remote locations where internet access is often limited, access to offline resources would be of benefit (i.e. educational materials).

Enhancing existing systems by increasing intensity of delivery and subsequent resources, may provide opportunities for improved support of recommended models of care and access to more integrated care [39]. Given the stakeholder representation on the Partnership, recommendations from this evaluation are likely to be integrated into current models of care. Consideration of linkages, relationships, interactions and behaviours of the complex system would promote further improvements [40]. Primary healthcare systems can be strengthened through systematic and partnership based approaches [41].

There were a number of limitations to our study. Invitations to focus groups were through professional networks which may not have reached all relevant health professionals and may have included individuals with an interest in the Partnership. Given this, results from focus groups are likely to have been positively skewed. Additionally, not capturing health professional’s time in their current position limited generalisability of references to high staff turnover for this group of participants. Furthermore, while the use of the SAT deviated from its intended use as a quantitative measure, it was useful in providing a framework for in-depth discussions in the focus groups (despite certain constructs not being discussed in all groups). Objective measurement was beyond the scope of this piece of work. Confounding challenges are that Aboriginal women’s understandings and expectations of DIP are largely unknown. A strength of the study is that views of a broad range of health professionals were captured across medical, nursing and allied health, representing multiple disciplines and government, Community-Controlled sectors, primary and tertiary and the third space. Furthermore, exclusion of patient and community perspectives may have missed other areas for improvement. The Partnership began by focusing on improving systems from health care professionals’ perspectives and funding has recently been secured to include the perspectives of women and patient engagement strategies in further work.

Nevertheless, this study seeks to improve systems and services for a high risk population in the context of challenges familiar to many on a global scale. The Global Alliance for Chronic Diseases has funded the scale-up of the NT DIP Partnership’s systems approach to other regions of Australia. This has the potential to inform further work in other settings, including Aboriginal populations internationally, as well as low and middle-income countries.

## Conclusion

The NT DIP Partnership has involved cross–sectoral collaboration into addressing gaps in models of care for women with diabetes in pregnancy in a complex setting. The service change will be an ongoing iterative cycle informed by the rich data in this process evaluation. Through a process evaluation focusing on perspectives of health professionals, we report quality improvement activities and enhanced models of care implemented since 2012, particularly through activities encouraging improved communication, education and use of guidelines. These processes facilitated health care providers self-reported confidence and ability to negotiate the care of women, along with improved models of care. While persisting challenges exist for delivery of care to a high risk population in remote Australia, the introduction of formal processes and structures, as demonstrated in this research, is likely to improve current capacity to deliver best practice care in other similarly challenging contexts [42].

## Abbreviations

ADIPS: Australasian Diabetes in Pregnancy Society
CQI: Continuous quality improvement
DIP: Diabetes in pregnancy
GDM: Gestational diabetes mellitus
HREC: Human Research Ethics Committee
MOC: Models of care
NT: Northern Territory
PANDORA: Pregnancy and Neonatal Outcomes in Remote Australia
SAT: Systems Assessment Tool
WHO: World Health Organisation
